# Nomogram for predicting reoperation following internal fixation of nondisplaced femoral neck fractures in elderly patients

**DOI:** 10.1186/s13018-021-02697-8

**Published:** 2021-09-01

**Authors:** Jian Zhu, Hongzhi Hu, Xiangtian Deng, Yiran Zhang, Xiaodong Cheng, Zhanchao Tan, Yanbin Zhu, Yingze Zhang

**Affiliations:** 1 School of Medicine, Nankai University, Tianjin, 300071 People’s Republic of China; 2 Department of Orthopedic Surgery, The Third Hospital of Hebei Medical University, Shijiazhuang, 050051 People’s Republic of China; 3 Key Laboratory of Biomechanics of Hebei Province, Orthopedic Research Institution of Hebei Province, Shijiazhuang, 050051 Hebei People’s Republic of China; 4 NHC Key Laboratory of Intelligent Orthopedic Equipment, The Third Hospital of Hebei Medical University, Shijiazhuang, 050051 Hebei People’s Republic of China; 5 Department of Orthopedics, Union Hospital, Tongji Medical College, Huazhong University of Science and Technology, Wuhan, 430022 People’s Republic of China

**Keywords:** Reoperation, Nondisplaced, Femoral neck fractures, Elderly, Nomogram

## Abstract

**Objective:**

We aimed to evaluate risk factors and develop a nomogram for reoperation after internal fixation of nondisplaced femoral neck fractures (FNFs) in elderly patients.

**Methods:**

We conducted a retrospective study involving a total of 255 elderly patients who underwent closed reduction and internal fixation with cannulated screw system for nondisplaced FNFs between January 2016 and January 2019. We collected data on demographics, preoperative radiological parameters, surgery, serum biochemical markers, and postoperative rehabilitation. In addition, we performed univariate and multivariate logistic regression analyses to determine independent risk factors for reoperation, and then developed a nomogram to assess the risks of reoperation. Besides, discriminative ability, calibration, and clinical usefulness of the nomogram were evaluated using the concordance index (C-index), the receiver operating characteristic (ROC) curve, calibration curve and decision curve analysis (DCA), respectively. We employed bootstrap method to validate the performance of the developed nomogram.

**Results:**

Our analysis showed that among the 255 patients, 28 (11.0%) underwent reoperation due to osteonecrosis of the femoral head (14 cases), mechanical failure (8 cases) or nonunion (6 cases). All of the 28 patients underwent conversion surgery to arthroplasty. The multivariate logistic regression analysis demonstrated that preoperative posterior tilt angle ≥ 20°, Pauwel’s III type, younger patients, preoperative elevated levels of alkaline phosphatase (ALP), preoperative hypoalbuminemia, and early postoperative weight-bearing were independent risk factors for reoperation. In addition, the C-index and the bootstrap value of the developed nomogram was 0.850 (95% CI = 0.803–0.913) and 0.811, respectively. Besides, the calibration curve showed good consistency between the actual diagnosed reoperation and the predicted probability, while the DCA indicated that the nomogram was clinically valuable.

**Conclusions:**

Our analysis showed we successfully developed and validated a nomogram for personalized prediction of reoperation after internal fixation of nondisplaced FNFs in elderly patients. This model would help in individualized evaluation of the need for reoperation and inform strategies aimed at eliminating the need for the reoperation.

## Introduction

Femoral neck fractures (FNFs) are common in geriatric population, accounting for 48 to 54% of hip fractures and 3.6% of the total fractures in adults [1]. Nearly 20% of all the FNFs are nondisplaced and often require surgical treatment [2, 3]. Owing to the simplicity, safety, efficacy, and affordability of closed reduction and internal fixation with cannulated screw system, it is widely used in the treatment of elderly patients with nondisplaced FNFs [2, 4]. However, previous reports have shown unsatisfactory clinical outcomes because of nonunion events, fixation failure or femoral head necrosis, which leads to patient readmission and reoperation [5–7]. The reoperation rate after internal fixation of nondisplaced FNFs in elderly patients ranged between 8 and 19% [8, 9]. Besides, reoperation is potentially devastating for the elderly patients, and the outcomes of salvage arthroplasty have been shown to be worse compared with those from the primary arthroplasty [10, 11]. Therefore, it is important to characterize reoperation and related predictors for better disease management.

Many studies have evaluated potential predictors of reoperation such as posterior tilt angle ≥ 20°, disruption of the medial cortex, higher level of fracture classification (Garden II or Pauwel’s III), advanced age, female patients, high score of Charlson Comorbidity Index (≥ 8), surgical delay, lower American Society of Anesthesiologists (ASA) grade, poor bone quality, and malnutrition [12–16]. However, serum biochemical markers and postoperative interventions were not included in the prediction system and thus were not integrated into the development of a nomogram. Therefore, there is need to develop an intuitive prediction model based on radiological measures, serum biochemical markers, and postoperative rehabilitative plans to inform treatment strategies.

Here, we evaluated multiple perioperative risk factors related to reoperation after internal fixation of nondisplaced FNF in elderly patients. Besides, we developed and validated a nomogram to intuitively predict personalized risks of reoperation to guide treatment options.

## Methods

### Patients

This study collected data from elder patients with nondisplaced FNFs and who underwent surgical treatment between January 2016 and January 2019. All the patients underwent closed reduction and internal fixation with cannulated screw system. The study followed the STROBE guidelines and was approved by the Institutional Review Board of the Third Hospital of Hebei Medical University (K2015-001-12). All the participants provided informed written consents.

### Inclusion and exclusion criteria

We included patients aged ≥ 65 years with a fresh nondisplaced FNF who had full baseline data. The patients were treated with closed reduction and internal fixation using a cannulated screw system and with a follow-up time ≥ 24 months. On the other hand, patients with pathological fractures and those with concomitant multiple fractures at the ipsilateral lower limb were excluded from the study.

### Surgical technique

Patient procedures were performed on a traction table without capsulotomies or hemarthrosis aspirations. After satisfactory reduction under fluoroscopy, three or four partially threaded, cancellous cannulated screws (6.5 mm; Stryker, Kalamazoo, MI, USA) were implanted percutaneously, in inverted triangle or rhombic configuration. The inferior screw was placed close to the femoral calcar, and all screws were inserted as deep as possible to purchase in the subchondral bone. After the operations, there was administration of intravenous cefazolin (2 g × 3 doses) for 24 h to prevent infection while low–molecular-weight heparin was used to prevent deep vein thrombosis (DVT). Besides, early weight-bearing was encouraged.

### Data collection

**Fig. 1:**
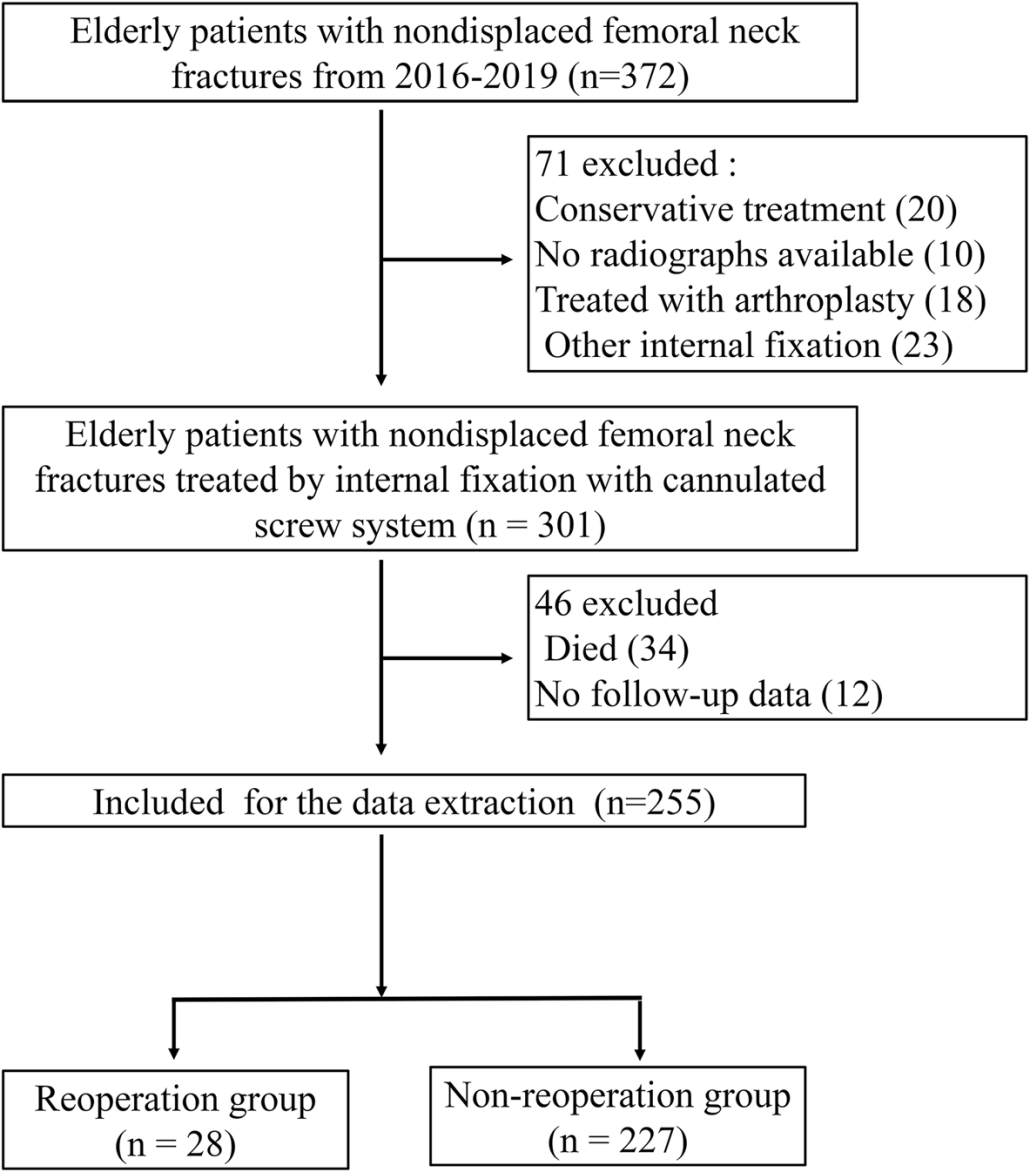
Patient selection flowchart

Baseline data were obtained from medical records, outpatient follow-ups, and questionnaire surveys. A total of 255 patients were included in our study (Fig. 1). We collected data on patient demographics, radiological parameters, surgery, serum biochemical markers, as well as postoperative rehabilitation.

The demographic data included gender, age, injured side, smoking status, alcohol consumption, preoperative functional status, cerebrovascular disease, hypertension, diabetes mellitus, Charlson’s weighted index of comorbidities (WIC), chronic kidney disease, and chronic obstructive pulmonary diseases (COPD). The Charlson’s WIC is the most commonly used method in the evaluation of the severity of comorbidities in elderly patients. It is calculated based on the patient’s medical history, prognosis, and weighted age [17]. Preoperative functional status was divided into either ambulatory aid or independent ambulator.

On the other hand, radiological parameters included Pauwel’s angle and posterior tilt angle. The posterior tilt angle was measured on the lateral radiographs and defined as the angle between the mid-collum line and the radius collum line [18]. The radiographic measurements were performed by two experienced orthopedists who were not involved in the operation. Surgery-related information included interval to surgery, type of anesthesia, the American Society of Anesthesiologists (ASA) classification, and surgical time. Interval to surgery was defined as the duration between fracture diagnosis and the onset of surgery.

Serum biochemical markers included red blood cell (RBC) count, white blood cell (WBC) count, platelet (PLT) count, lymphocyte (LYM) count, hemoglobin (HGB) level, alanine transaminase (ALT), aspartate transaminase (AST), alkaline phosphatase (ALP), sodium concentration (Na^+^), total protein (TP) level, albumin (ALB) level, and D-dimer level. Information on postoperative rehabilitation included interval to weight-bearing. The definition of weight-bearing was that patients began to ambulate with bilateral crutches or walker assistance. In addition, clinical outcomes at last follow-up, such as the Harris hip score and visual analogue scale (VAS), were also recorded.

Reoperation was defined as any operation performed due to complication after the primary internal fixation [19]. The complication included mechanical failure, nonunion of fracture, or avascular necrosis of the femoral head.

### Statistical analysis

SPSS 26 software (IBM Corp., Armonk, NY, USA) was used for data analysis while R software (version 3.6.5, R Foundation for Statistical Computing, Vienna, Austria) with the “rms” package was used for nomogram construction. The interobserver reliability was measured using the kappa coefficient (*κ*) for Pauwel’s classification and intraclass correlation coefficient (ICC) for posterior tilt angle. The normality of population was evaluated using the Shapiro–Wilk test. Continuous data were presented as mean ± standard deviation (SD) or median and interquartile range as evaluated with the Student *t* test or Mann–Whitney *U* test based on the data distribution. On the other hand, categorical data were presented as numbers (%) and then compared with chi-squared or Fisher’s exact, as appropriate. The variables with *P* < 0.10 in the univariable analyses were analyzed in a multivariable logistic regression to identify independent risk factors of reoperation. Thereafter, a nomogram was constructed using the independent risk factors obtained from the multivariable logistic regression analysis. The predictive ability and performance of the model was assessed with the concordance index (C-index), the receiver operating characteristic (ROC) curve, calibration curve, as well as decision curve analysis (DCA). The C-index and the area under the ROC curve (AUC) were used to evaluate the predictive accuracy and discriminative ability of the nomogram [20]. The value of the C-index ranged between 0.5 and 1.0, where a larger value indicated more accuracy of the nomogram in distinguishing the subjects [21]. The relationship between the actual diagnosed reoperation and the predicted probability of the reoperation was evaluated with a calibration curve. In addition, the clinical usefulness of the nomogram was evaluated with the DCA based on the net benefit and threshold probabilities. A *P* value < 0.05 was considered to be statistically significant. Finally, a corrected C-index was calculated through bootstrapping (1000 resamples) to evaluate the accuracy of the nomogram.

## Results

### Patient baseline data

A total of 255 elderly patients with nondisplaced FNF were included in our study. Our analysis showed that the mean follow-up duration was 42.7 (24–60) months. The patients included 64 males and 191 females, with an average age of 73.2 years (range, 65–92). Among them, 28 patients experienced reoperation, and the most common reason was osteonecrosis of the femoral head (14 cases), followed by mechanical failure (8 cases) and nonunion (6 cases). None of the patients experienced deep infection or subtrochanteric fracture. All of the 28 patients underwent conversion surgery to arthroplasty.

### Univariate and multivariate analysis

The interobserver reliability of the radiographic characteristics (Pauwel’s classification and posterior tilt angle) was evaluated, and then initial demographical and perioperative variables were compared between the reoperation and non-reoperation groups (Tables 1 and 2). The data showed significant differences in age (*P* = 0.015), posterior tilt angle (*P* = 0.023), AST (*P* = 0.045), ALP (*P* = 0.012), ALB (*P* = 0.025), interval to weight-bearing (*P* = 0.001), Harris hip score (*P* = 0.004), and VAS (*P* = 0.016). According to Pauwel’s classification, the most common fracture pattern in the reoperation group was Pauwel’s III (*n* = 12, 42.9%), followed by Pauwel’s II (*n* = 9, 32.1%) and Pauwel’s I (*n* = 7, 25.0%). On the other hand, Pauwel’s II (*n* = 94, 41.4%) was the most common fracture pattern in the non-reoperation group, followed by Pauwel’s I (*n* = 86, 37.9%) and Pauwel’s III (*n* = 47, 20.7%), with a significant difference in the fracture pattern distribution (*P* = 0.031). Based on the *P* < 0.10 threshold, all these risk factors and Na^+^ (*P* = 0.080) were analyzed in the multivariable logistic regression to identify the independent risk factors for reoperation.

**Table 1:** Interobserver reliability of the radiographic characteristics

Characteristics	ICC or κ	95% CI	*P* value
Pauwel’s classification	0.915	0.870 to 0.960	< 0.001
Posterior tilt angle	0.885	0.854 to 0.909	< 0.001

Abbreviations: *ICC* intraclass correlation coefficient, *κ* kappa coefficient, *CI* confidence interval

**Table 2:** Characteristics of reoperation and non-reoperation patients

Characteristics	Reoperation, *n* = 28 (%)	Non-reoperation, *n* = 227 (%)	*P* value
Gender			0.362
Male	9 (32.1)	55 (24.2)	
Female	19 (67.9)	172 (75.8)	
Age	68.0 (67.0–73.0)	72.0 (67.0–78.0)	0.015^*^
Injured side			0.401
Right	10 (35.7)	100 (44.1)	
Left	18 (64.3)	127 (55.9)	
Smoking			0.549
No	25 (89.3)	210 (92.5)	
Yes	3 (10.7)	17 (7.5)	
Alcohol consumption			0.302
No	26 (92.9)	219 (96.5)	
Yes	2 (7.1)	8 (3.5)	
ASA classification			0.411
I–II	23 (82.1)	199 (87.7)	
III–IV	5 (17.9)	28 (12.3)	
Chronic kidney disease			0.435
No	26 (92.9)	218 (96.0)	
Yes	2 (7.1)	9 (4.0)	
Hypertension			0.695
No	11 (39.3)	98 (43.2)	
Yes	17 (60.7)	129 (56.8)	
Diabetes mellitus			0.205
No	24 (85.7)	170 (74.9)	
Yes	4 (14.3)	57 (25.1)	
Charlson’s WIC	3.54 ± 1.26	3.70 ± 1.35	0.541
Cerebrovascular disease			0.425
No	23 (82.1)	171 (75.3)	
Yes	5 (17.9)	56 (24.7)	
COPD			0.217
No	24 (85.7)	210 (92.5)	
Yes	4 (14.3)	17 (7.5)	
Preoperative functional status			0.713
Using ambulatory aid	2 (7.1)	21 (9.3)	
Independent ambulator	26 (92.9)	206 (90.7)	
Interval to surgery			0.335
< 72 h	14 (50.0)	81 (35.7)	
72 - 120 h	7 (25.0)	72 (31.7)	
> 120 h	7 (25.0)	74 (32.6)	
Pauwel’s classification			0.031^*^
I	7 (25.0)	86 (37.9)	
II	9 (32.1)	94 (41.4)	
III	12 (42.9)	47 (20.7)	
Posterior tilt angle			0.023^*^
≥ 20°	13 (46.4)	59 (26.0)	
< 20°	15 (53.6)	168 (74.0)	
Anesthesia type			0.168
General anesthesia	9 (32.1)	47 (20.7)	
Regional anesthesia	19 (67.9)	180 (79.3)	
Surgical time (min)	75.89 ± 39.77	80.68 ± 29.64	0.439
WBC (>10 × 10^9^ /L)	2 (7.1)	35 (15.4)	0.392
RBC (< lower limit) ^a^	8 (28.6)	49 (21.6)	0.403
PLT (> 300 × 10^9^ /L)	2 (7.1)	14 (6.2)	0.841
LYM (< 1.1 × 10^9^/L)	10 (35.7)	80 (35.2)	0.961
HGB (< lower limit) ^b^	10 (35.7)	51 (22.5)	0.121
ALT (> 40 U/L)	4 (14.3)	16 (7.0)	0.251
AST (> 40 U/L)	4 (14.3)	11 (4.8)	0.045^*^
ALP (>125 U/L)	7 (25.0)	21 (9.3)	0.012^*^
Na^+^ (< 135 mmol/L)	7 (25.0)	29 (12.8)	0.080
TP (< 60 g/L)	8 (28.6)	41 (18.1)	0.183
ALB (< 35 U/L)	8 (28.6)	29 (12.8)	0.025^*^
D-dimer (> 0.5 mg/L)	17 (60.7)	151 (66.5)	0.541
Interval to weight-bearing	6.39 ± 2.74	8.24 ± 2.73	0.001^*^
Harris Hip Score	76.68 ± 7.69	81.59 ± 8.60	0.004*
VAS	2.57 ± 1.60	1.78 ± 1.30	0.016*

Abbreviations: *ASA* the American Society of Anesthesiologists, *WIC* Charlson’s weighted index of comorbidities, *COPD* chronic obstructive pulmonary diseases, *WBC* white blood cell, *RBC* red blood cell, *PLT* platelet, *LYM* lymphocyte, *HGB* hemoglobin, *ALT* alanine transaminase, *AST* aspartate transaminase, *ALP* alkaline phosphatase, *Na+* serum sodium concentration, *TP* total protein, *ALB* albumin

*: Statistically significant difference

a Reference range: women 3.5–-5.0 × 10^12^/L, men 4.0–-5.5 × 10^12^/L

b Reference range: women 110–-150 g/L, men 120–-160 g/L

The multivariate analysis demonstrated that age (odds ratio (OR) = 0.910, 95% confidence interval (CI) = 0.837–0.989, *P* = 0.026), posterior tilt angle (OR = 2.986, 95% CI = 1.143–7.797, *P* = 0.026), ALP (OR = 4.033, 95% CI = 1.275–12.756, *P* = 0.018), ALB (OR = 5.345, 95% CI = 1.577–18.116, *P* = 0.007), Pauwel’s III classification (OR = 5.056, 95% CI = 1.498–17.062, *P* = 0.009), and interval to weight-bearing (OR = 0.739, 95% CI = 0.618–0.885, *P* = 0.001) were independent risk factors for reoperation after internal fixation of nondisplaced FNFs in elderly patients (Table 3).

**Table 3:** Multivariate analysis of risk factors associated with reoperation after internal fixation of nondisplaced FNFs in elderly patients

Characteristics	OR	95% CI (lower limit)	95% CI (upper limit)	*P* value
Age	0.910	0.837	0.989	0.026^*^
Posterior tilt angle	2.986	1.143	7.797	0.026^*^
AST (> 40 U/L)	3.026	0.676	13.532	0.147
ALP (> 125 U/L)	4.033	1.275	12.756	0.018^*^
Na^+^ (< 135 mmol/L)	2.875	0.902	9.163	0.074
ALB (< 35 U/L)	5.345	1.577	18.116	0.007^*^
Interval to weight-bearing	0.739	0.618	0.885	0.001^*^
Pauwel’s classification				
I	Ref.			
II	1.915	0.508	7.217	0.337
III	5.056	1.498	17.062	0.009^*^

Abbreviations: *OR* odds ratio, *CI* confidence interval, *AST* aspartate transaminase, *ALP* alkaline phosphatase, *Na+* serum sodium concentration, *ALB* albumin

*: Statistically significant difference

### Development and validation of a reoperation nomogram

**Fig. 2:**
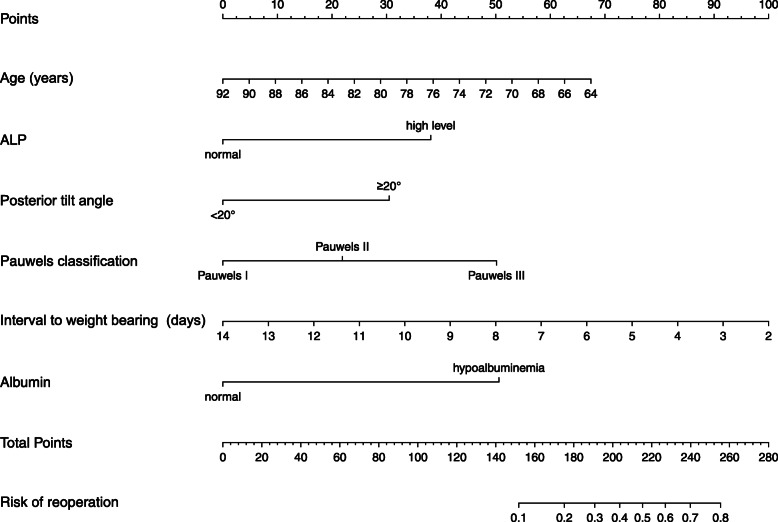
Nomogram for predicting reoperation in elderly patients with nondisplaced FNFs. *ALP* alkaline phosphatase, *ALB* albumin

**Fig. 3:**
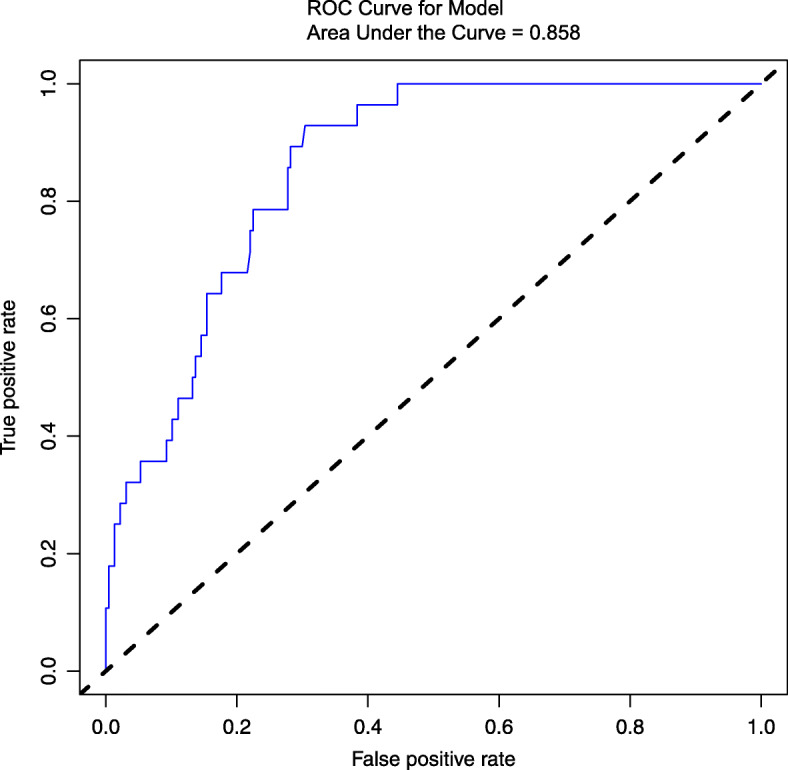
The ROC analysis for the predictive model

**Fig. 4:**
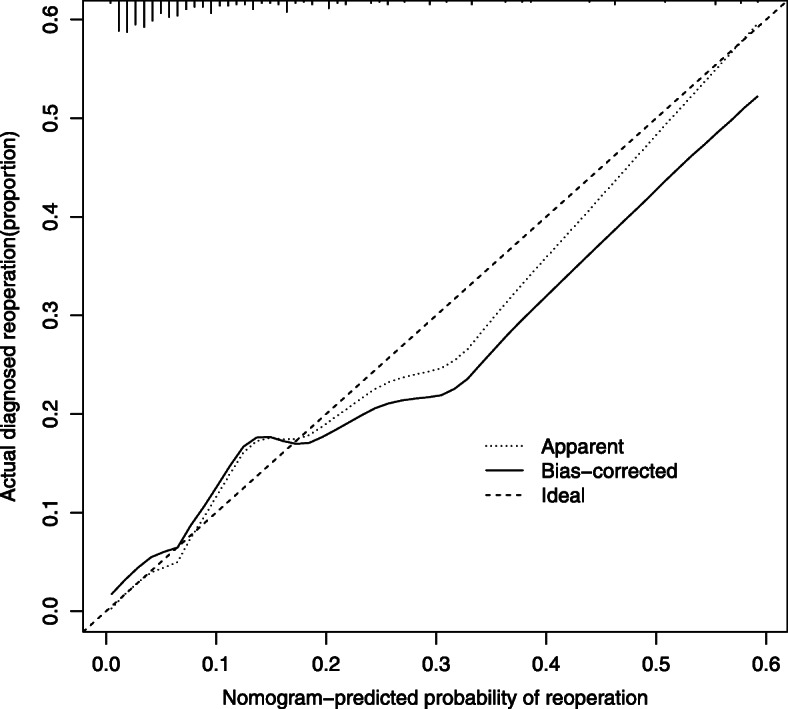
The calibration curve indicated good consistency between the actual diagnosed reoperation and the predicted probability.

**Fig. 5:**
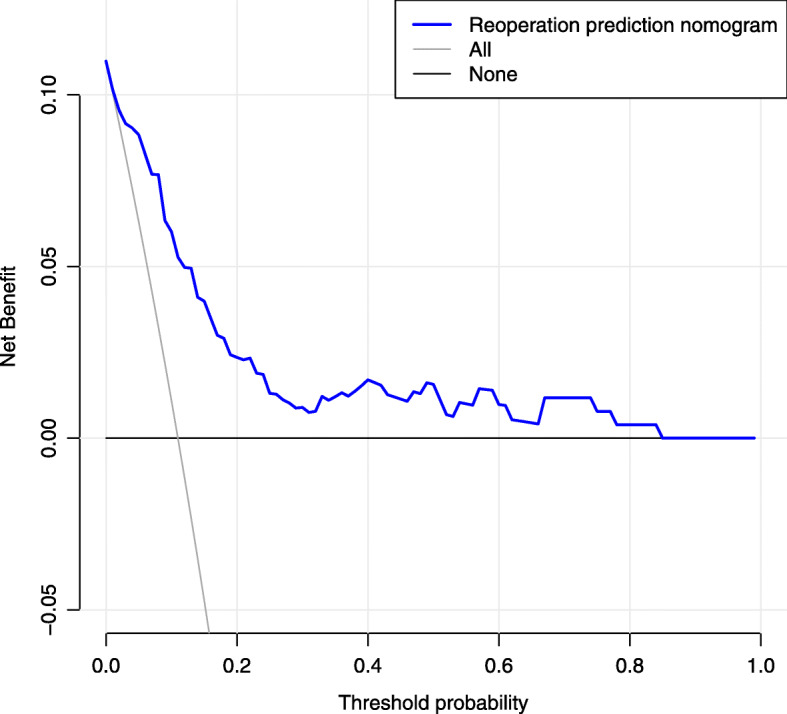
Decision curve analysis for the reoperation nomogram

Using the independent risk factors obtained from the multivariable logistic regression analyses, we constructed a nomogram to predict reoperation (Fig. 2). By adding individual scores of each predictor in the nomogram, the total score was obtained and used to calculate the corresponding reoperation probability. This was helpful for accurate evaluation of preoperative risks and determination of the reoperation cases. Our analyses showed high predictive accuracy and discrimination of the model, with a C- index of 0.850 (95% CI = 0.803–0.913) and an AUC of 0.858 (Fig. 3). In addition, the corrected C-index was 0.811 in the bootstrapping validation. Besides, the calibration curve of the nomogram demonstrated good consistency between the actual diagnosed reoperation and the predicted probability (Fig. 4). Similarly, the nomogram DCA indicated that the model could be an excellent prediction tool for reoperation after internal fixation of nondisplaced FNFs in elderly patients (Fig. 5).

## Discussion

In this study, we analyzed a total of 255 elderly patients to develop and validate a predictive clinical nomogram for reoperation after closed reduction and internal fixation of FNFs. Our findings showed that the reoperation rate within at least a 2-year follow-up was 11.0%, which was consistent with previous reports [8, 9]. Six key risk factors were included in the nomogram: preoperative posterior tilt angle, Pauwel’s classification, age, preoperative ALP, preoperative ALB, and interval to weight-bearing.

Reoperation after internal fixation of nondisplaced FNF in elderly patients could be precipitated by several factors, with posterior tilt angle having the highest prognostic value [12, 15, 22]. Currently, at least two mechanisms have been identified as main reasons for reoperation associated with preoperative posterior tilt angle. Firstly, the greater posterior tilt is associated with more posterior comminution of the femoral neck [22], which compromises the stability of internal fixation. Honkanen et al. [23] showed similar reoperation rate between nondisplaced FNF patients with ≥ 20° posterior tilt angle and those with displaced FNF. Thus, the posterior tilt on lateral radiograph is as vital as displacement on the anteroposterior radiograph. Similarly, irreversible damage to the retinacular vessels which could result in either nonunion in the early stage or femoral head necrosis in the later stage has also been associated with reoperation [18]. In case of FNF with either varus angulation on the anteroposterior view or posterior tilt on the lateral view, the fragile lateral epiphyseal arteries could be torn or kinked, which would devastate the femoral head vitality [24]. Consistent with previous studies, our data showed that posterior tilt angle ≥ 20° could predict reoperation in elderly patients with FNFs. Therefore, we suggest thorough evaluation of the preoperative lateral radiograph of hip joint to identify the patients with high posterior tilt angle.

In addition, Pauwel’s classification is important and widely accepted in guiding treatment and rehabilitation of FNF [25]. In the present study, we analyzed the reoperation risk of patients with different Pauwel’s classification. Both univariate and multivariate analyses showed that higher Pauwel’s levels implied higher reoperation risks, which was in agreement with the finding obtained by Biz et al. [26]. They showed that the rates of internal fixation failure in patients with nondisplaced FNF had significant differences between Pauwel’s II or III group and Pauwel’s I group. In parallel, our study showed that only Pauwel’s III FNF could predict reoperation. This could be because a vertical fracture line of Pauwel’s III FNF is subjected to higher shear force and prone to inferior translation, and thus, early weight-bearing after surgery could challenge the stability of implants and lead to failure of internal fixation [27, 28]. Together, we demonstrate that, compared with internal fixation, elderly FNF patients with a Pauwel’s III classification would benefit from primary arthroplasty by avoiding reoperation.

Although the effect of patient’s age on reoperation after internal fixation of FNF in the elderly is important, the available data remains controversial. While other studies have demonstrated that advanced age is related with increase in reoperation [15, 29], others could not find any correlation between the age of the patients and the rate of reoperation [30, 31]. In contrast, our study showed that the younger patients in the elderly group were at a higher risk of revision surgery. This could be due to the fact that the younger individuals were not as frail and functionally impaired as the older ones; therefore, they were more sensitive to the complications of internal fixation. Indeed, a previous report by Rogmark et al. [31] demonstrated that younger patients in the elderly group had a higher frequency of reporting subjective pain after internal fixation compared with those with advanced age (51% vs 27%, *p* = 0.016). In addition, the elderly patients with advanced age had a higher mortality rate compared with the younger ones, which could lead to underestimation of the number of possible reoperations [30], and thus might have influenced our findings.

High ALP levels were shown to predict reoperation in elderly patients with nondisplaced FNF in our study. Since the ALP isoenzymes in serum could emanate from the liver, bone, or intestine, there is need to differentiate the elevated ALP from bone tumor, osteomalacia or hepatic diseases. Our study showed that the high levels of ALP were associated with reoperation and not any potential comorbidity. A potential cause might be that a lower BMD was also present in these patients. As a main biochemical marker of bone turnover, ALP is related with bone formation and mineralization. Tariq et al. [32] reported that ALP could be used to predict the BMD in postmenopausal females, and elevated ALP was associated with loss of BMD. In their study, Zhao et al. [33] showed a negative correlation between the ALP level and the BMD value of the femoral neck in patients with osteoporosis. Besides, it has been shown that the bone quality in elderly people plays an important role in the outcome of internal fixation [4]. In a biomechanical experiment, Sjöstedt et al. [34] showed that low BMD could weaken the strength of fixation. Hence, as an independent risk factor for reoperation, the ALP level should be integral in the evaluation of reoperation risk in the elderly patients with FNFs. On the other hand, low albumin level has been reported to be a significant risk factor for reoperation [12, 35]. However, data on the exact reason remains scant. Previous studies showed lower albumin level was associated with low BMD and recurrent falls after low-energy injury [36, 37]. In addition, we hypothesize that euthyroid sick syndrome (ESS) might be responsible for reoperation in patients with hypoalbuminemia. It has been shown that hypoalbuminemia correlated with ESS in elderly patients [38]. In recent studies, ESS was reported to correlate with perioperative anemia and metabolic disorder of Vitamin D, as well as parathyroid hormone (PTH) [39, 40], which could increase the possibility of reoperation after hip fractures [41, 42]. However, due to the retrospective nature of our study, we could not evaluate the ESS due to missing data on the thyroid hormone profiles. Thus, prospective studies are required to determine the association between ESS and reoperation. Together, we suggest assessment of serum ALP and ALB alongside radiological measures for effective determination of the optimal surgical strategy: arthroplasty or internal fixation.

To date, there is lack of ideal protocol for weight-bearing after internal fixation of nondisplaced FNF in elderly patients. Schwachmeyer et al. [43] suggested that patients with hip surgery should avoid early weight-bearing after analyzing the hip contact loads in rehabilitation exercises. On the other hand, Kim et al. [44] showed that elderly FNF patients with full postoperative weight-bearing at an average of 5.2 days had excellent clinical and functional outcome. In our study, we showed that early weight-bearing after surgery would increase the incidence of reoperation. Besides increasing the risk of failure of internal fixation, early weight-bearing could result in the occurrence reoperation through the following mechanisms. Firstly, owing to decreased bone quality in the elderly patients and bone resorption at the fracture site, early weight-bearing could lead to shortened femoral neck, which is a significant risk factor for osteonecrosis of the femoral head [45]. Secondly, since there is poor body control and insufficient arm strength in the elderly patients, it is difficult for them to perform partial weight-bearing exercise postoperatively. Early full weight-bearing would increase the intra-capsular pressure and result in high risk of osteonecrosis [46]. Our data showed that the elderly nondisplaced FNF patients would benefit from delayed weight-bearing postoperatively. However, the benefit of the delayed weight-bearing in reducing the reoperation rate must be balanced against potential complications due to bed rest, such as thrombosis, pneumonia, and increased mortality. Therefore, there should be a patient-tailored postoperative rehabilitation strategy to decrease the risks for reoperation after FNF based on preoperative fracture pattern, serum biochemical markers, and the patients’ general condition.

Having evaluated the potential risk factors such as radiological parameters, serum biochemical markers, and postoperative rehabilitation, we developed a nomogram for prediction of reoperation. The nomogram could inform individualized evaluation of reoperation risks in elderly patients who undergo internal fixation. Accordingly, primary arthroplasty could be used in patients with a high risk of reoperation as predicted by the constructed model. Furthermore, patients with high predictive value due to higher posterior tilt or Pauwel’s angle could reduce the probability of reoperation by improving the postoperative variables such as weight-bearing.

Our study successfully developed and validated a nomogram for prediction of reoperation following internal fixation of nondisplaced FNF in elderly patients. The nomogram is visual and user-friendly. However, our study was conducted retrospectively, and thus inherent selection bias might have affected our findings. Besides, the fixation operations were not performed by the same surgeon, which could affect the surgical outcomes. Some patients had indication for reoperation but could not be performed due to their general condition and fragility, which might have led to underestimation of the incidence of reoperation. In addition, since the nomogram was only validated internally, there is need for further external validation.

## Conclusion

In summary, we successfully developed and validated a nomogram model for individualized prediction of reoperation after internal fixation of nondisplaced FNF in elderly patients based on perioperative variables. Based on the nomogram model, primary arthroplasty should be considered rather than internal fixation for elderly patients with high predictive values.

## Data Availability

The data contributing to this article may be made available upon request by sending an e-mail to the first author.

## Abbreviations

FNF: Femoral neck fractures
ASA: American Society of Anesthesiologists
WIC: Charlson’s weighted index of comorbidities
COPD: Chronic obstructive pulmonary diseases
WBC: White blood cell
RBC: Red blood cell
PLT: Platelet
LYM: Lymphocyte
HGB: Hemoglobin
ALT: Alanine transaminase
AST: Aspartate transaminase
ALP: Alkaline phosphatase
Na^+^: Serum sodium concentration
TP: Total protein
ALB: Albumin
VAS: Visual analogue scale
κ: kappa coefficient
ICC: Intraclass correlation coefficient
ROC: Receiver operating characteristic
C-index: Concordance index
DCA: Decision curve analysis
AUC: Area under receiver operating characteristic curve
OR: Odds ratio
CI: Confidence interval
BMD: Bone mineral density
ESS: Euthyroid sick syndrome
